# Commentary: T cell metabolism: a new perspective on Th17/Treg cell imbalance in systemic lupus erythematosus

**DOI:** 10.3389/fimmu.2023.1164761

**Published:** 2023-04-25

**Authors:** Jiamei Jin, Xiaogu Liu

**Affiliations:** School of Basic Medicine, Zhejiang Chinese Medical University, Hangzhou, China

**Keywords:** cell metabolism, T helper 17 cells, regulatory T cells, systemic lupus erythematosus, fatty acid oxidation

## 1 Introduction

A study entitled “*T cell metabolism: A new perspective on Th17/Treg cell imbalance in systemic lupus erythematosus*” was published in *Frontiers in Immunology* by Shan J and his colleagues (1). The article’s summary claims that Th17/regulatory T cells (Treg) play a role in the initiation and progression of systemic lupus erythematosus (SLE). Mounting evidence suggests that metabolic pathways participate in inflammatory responses in SLE by regulating T-cell differentiation and function. A novel SLE treatment reverses the Th17/Treg imbalance by targeting metabolic pathways. SLE patients have distinct T-cell metabolic patterns, such as glycolysis, lipid synthesis, and glutaminolysis, all of which are conducive to Th17 cell differentiation and function. Conversely, Tregs rely more on energy from oxidative metabolism and fatty acid oxidation.

Under the subheading of *Metabolic control of Th17/Treg balance*, this article first described “Tregs, on the other hand, were found to rely more on fatty acid oxidation and oxidative phosphorylation to supply energy” and then described “Conversely, inhibition of fatty acid oxidation results in diminished differentiation of Th17 cells but increased development of Tregs.” There is an apparent inconsistency between the two accounts.

Two references cited here show that Tregs not only oxidize lipids at a high rate, but also oxidize pyruvate derived from glycolysis. Specifically, Teffs (Th1 and Th17) depend on glycolysis, whereas Tregs have higher flexibility in fuel choice and can oxidize glucose in addition to fats (2). Moreover, Tregs and memory CD8+ cells preferably rely on mitochondrial fatty acid oxidation to meet cell energy requirements; their energy production pathways are binary: oxidative fatty acid uptake and glycolysis/glutamine decomposition, respectively (3). Numerous studies have shown that Tregs depend on fatty acid oxidation for energy supply, and inhibition of fatty acid oxidation can hinder Treg development (4–8). Hence, the words “diminished” and “increased” should switch places in the description above.

Further studies are needed to investigate T cells’ metabolic abnormalities in SLE patients, their role in disease progression, and their response to therapy, especially activities interfering with the Th17/Treg cell imbalance. We believe that the summary by Shan J and his colleagues will help us better understand the relationship between metabolic abnormalities in T cells and the Th17/Treg balance. However, this review needs to be written more rigorously to demonstrate that the metabolic profile of T cells underlies the Th17/Treg cell imbalance in SLE patients.

## Author contributions

JJ conceived the original idea and wrote the manuscript with support from XL. All authors listed have made a substantial, direct, and intellectual contribution to the work and approved it for publication.
